# Red blood cell count has an independent contribution to the prediction of ultrasonography-diagnosed fatty liver disease

**DOI:** 10.1371/journal.pone.0172027

**Published:** 2017-02-10

**Authors:** Hai-lin Wang, Hui Zhang, Shang-ling Wu, Gong-cheng Liao, Ai-ping Fang, Ming-fan Zhu, Hui-lian Zhu

**Affiliations:** 1 Faculty of Nutrition, School of Public Health, Sun Yat-sen University, Guangzhou, Guangdong Province, The People’s Republic of China; 2 Health Examination Centre, Shenzhen Luohu People’s Hospital, Shenzhen, Guangdong Province, The People’s Republic of China; 3 Health Examination Centre, First Affiliated Hospital of Sun Yat-sen University, Guangzhou, Guangdong Province, The People’s Republic of China; Chang Gung Memorial Hospital Kaohsiung Branch, TAIWAN

## Abstract

**Background & aims:**

Red blood cell (RBC) indices have been demonstrated to be associated with fatty liver disease (FLD) and metabolic syndrome. However, controversy exists regarding the relationship of RBC indices with FLD to date and few has focused on RBC count. This study aimed to explore the association between RBC count and risk of FLD in Southern Chinese adults.

**Methods:**

A hospital-based cross-sectional study was performed in two hospital health examination centers, including information on ultrasonography-diagnosed FLD, anthropometric indices and biochemical measurements. Covariance analysis was used to evaluate group differences. After quintile classification of RBC counts, logistic regression analysis was conducted to evaluate the odds ratios (ORs) of FLD.

**Results:**

This study consisted of 8618 subjects (4137 men and 4481 women) aged between 20 and 89 years. FLD cases had higher RBC counts than non-FLD cases in both genders (P<0.001). The prevalence rates of FLD increased with the RBC quintiles in both genders (all P trend<0.001), and were higher in men than women. Binary logistic regression analysis showed positive association between RBC count and FLD, and the OR (95% confidence interval (CI)) were 2.56 (2.06–3.18) in men and 3.69 (2.74–4.98) in women, respectively, when comparing Q5 with Q1. Stratified analyses showed similar trends among subjects with and without FLD risk factors. Gender independent results were similar to gender dependent results.

**Conclusions:**

Elevated RBC count is independently associated with high risk of FLD, suggesting that the RBC count may be a potential risk predictor for FLD.

## Introduction

Fatty liver disease (FLD) is currently emerging as the most common cause of chronic liver disease throughout the world, with a prevalence of 20%-40% in Western countries and 15%-35% in different regions of China [1–3]. Ranging from simple steatosis and steatohepatitis to liver fibrosis, FLD has the potential risk to progress to end-stage liver diseases, such as liver cirrhosis, hepatocellular carcinoma, and possible liver failure. FLD is widely regarded as the hepatic manifestation of metabolic syndrome and is closely associated with obesity, type 2 diabetes, dyslipidemia, and an increased level of serum fatty acids. The widely accepted pathogenesis of FLD is a ‘two-hit’ hypothesis, which is related to the mechanisms of inflammation and oxidative stress [4, 5].

Under physiological conditions, red blood cells (RBCs) are responsible for not only delivering oxygen and nitric oxide to the periphery and carbon dioxide to the lungs but also scavenging reactive oxygen and nitrogen species generated in some tissues [6, 7]. The liver plays a major role in metabolism, has numerous functions in the human body and intensively produces pro-oxidant reactive species. When RBCs flow through liver tissue and are unable to counteract oxidation status, they become a source of reactive oxygen species (ROS). The liver is also responsible for eliminating senescent RBCs by residential macrophages [8], leading the release of iron, triggering oxidative stress in liver tissue. Therefore, higher RBC count might promote oxidative stress and exacerbate a fatty liver [9].

Previous studies have suggested that RBCs are associated with metabolic syndrome and insulin resistance. A four-year follow-up longitudinal cohort study with 6,453 participants found that red blood cells were strongly associated with metabolic syndromes (MetS) (RR: 3.016; 95% CI: 1.525–5.967; P value: 0.002) and showed positive trends with obesity, hypertension and dyslipidemia [10]. Barbieri surveyed 608 participants and found that RBCs are positively associated with HOMA, insulin and markers related to metabolic syndrome (triglycerides, low-density lipoprotein cholesterol and body mass index) [11, 12]. Other studies also supported that high levels of red blood cells could increase the risk of metabolic syndrome [13–17]. However, only a few studies have investigated the relationship between the RBC count and FLD [12, 18], which is the manifestation of metabolic syndrome in the liver, but the results are inconsistent. Therefore, we conducted a large-scale cross-sectional study to explore the independent relationship between the RBC count and risks of FLD in southern Chinese adults, which might provide some novel insights regarding the early diagnosis of FLD.

## Materials and methods

### Participants

This cross-sectional study was conducted among participants from the Health Examination Centers of the First Affiliated Hospital of Sun Yat-sen University between May 2009 and April 2010 (Guangzhou, China, n = 4771) and Shenzhen Luohu People’s Hospital between April 2014 and March 2015 (Shenzhen, China, n = 5005). Subjects meeting one of the following criteria were excluded: (1) participants less than 20 years of age; (2) participants taking antihypertensive agents, antidiabetic drugs, or lipid-lowering agents; (3) subjects with a history of malignancy, thyroid diseases, other known causes of chronic liver disease such as viral hepatitis or autoimmune hepatitis; and (4) subjects who had missing data related to RBC count or ultrasonography that confirmed FLD. Eventually, a total of 8618 eligible subjects (4137 men, 4481 women) were enrolled for analysis. The study protocol was approved by the Ethics Committees of Sun Yat-sen University, and written informed consent was acquired from every participant.

### Clinical and anthropometric measurements

A medical questionnaire was used to collect general information from each participant. Physical examinations were administered to all subjects by experienced physicians in the morning after an overnight fast, and participants were informed to avoid strenuous exercise during the day before their examinations. With subjects bare-footed and wearing light clothing, standing height and body weight were measured with a portable stadiometer (Holtain, Crymmych, Wales), accurate to 0.1 cm, and a digital scale (Hanson, Watford, Hertforshire, England), accurate to 0.1 kg, respectively. Body mass index (BMI, kg/m^2^) was calculated as weight in kilograms divided by height in square meters. Waist circumference (WC) was measured at the midpoint between the lowest rib and superior border of the iliac crest, and hip circumference was measured at the greater trochanter with measuring tapes accurate to 1 mm. The waist-to-hip ratio (WHR) was calculated as the waist circumference divided by the hip circumference. Blood pressure was measured at the upper right arm using a calibrated sphygmomanometer (Hawksley, WA Baum Co, USA) with the subject in the sitting position after 15 minutes of rest.

Overweight was defined as BMI ≥ 24 kg/m^2^ for both genders [19], and central obesity was defined as WC ≥ 80 cm in women and ≥ 90 cm in men [20]. Impaired fasting glucose (IFG) was defined as fasting blood glucose (FBG) ≥ 5.6 mmol/L, and diabetes was defined as FPG levels ≥ 7.0 mmol/L or a self-reported history of diabetes confirmed by the doctor [21].

### Biochemical analyses

Fasting blood samples were collected from the antecubital vein and used for analyses of biochemical indices in the biochemistry laboratory of the hospital. Total cholesterol (TC) and triglyceride (TG) concentrations were determined using an enzymatic colorimetric test. The selective inhibition method and homogeneous enzymatic colorimetric test (Advia1650 Autoanalyzer, Byer Diagnostics, Leverkusen, Germany) were used to measure the levels of high-density lipoprotein cholesterol (HDL-C) and low-density lipoprotein cholesterol (LDL-C), respectively. Alanine transaminase (ALT), aspartate transaminase (AST) and γ-glutamyl transpeptidase (GGT) were determined with an automated analyzer (Technicon Sequential Multiple Analyzer, Technicon Instruments Corporation, Tarrytown, NY). Fasting blood glucose (FBG) was measured in venous blood. RBC count, white blood cell count, platelet count, and hemoglobin levels were measured using an automatic hematology analyzer (Roche, Cedex, Gemany).

### Ultrasonography

Using a scanner (X200, Toshiba Corporation, Japan), a real-time ultrasound examination of the upper abdominal organs was performed in all participants by two well-trained ultrasonographers who were blinded to the clinical and laboratory findings. FLD was diagnosed and semi-quantified on the basis of the abdominal ultrasound results combined with medical histories, clinical symptoms and laboratory results of the subjects, which was in accord with the method used by M. Graif and later adopted by the Chinese Society of Hepatology [22, 23]. The diagnosis of FLD was transformed into binary categories (1 and 0 denoted FLD and Non-FLD, respectively).

### Statistical analyses

Statistical analyses were performed using the SPSS version 20.0 for Windows (IBM, Inc., Armonk, New York, USA). All data were analyzed with and without gender stratification. Continuous variables were tested for normality, and the skewed variables (ALT, AST and GGT) were normalized using logarithmic transformation. Continuous variables were expressed as the means and standard deviations (Means ± SD) or the medians and interquartile ranges according to their normal distribution status. Frequencies and percentages were presented for categorical data. After adjusting for age, covariance analysis was used to compare the mean differences between the FLD and non-FLD groups, and the Wald chi-square test was applied to explore proportional differences. The RBC count was classified into quintiles (Q1-Q5), with Q1 used as the reference. Logistic stepwise regression analysis was applied to determine the relationship between the RBC level and prevalence of FLD. A two-tailed test with P<0.05 was considered statistically significant.

## Results

Among the 8618 enrolled participants, the mean (±SD) age was 43.04±11.99 years and 4137 (48.0%) of the participants were men. Of all subjects, 2124 of the participants (24.65%, 1455 men and 669 women) met the ultrasound diagnostic criteria for FLD. The demographic, clinical, and metabolic characteristics of all participants are displayed in Table 1. The prevalence of FLD was 35.17% and 14.93% in men and women, respectively. FLD subjects tended to be older than Non-FLD individuals in both men (P = 0.012) and women (P<0.001). After stratification by gender and adjusting for age, the FLD patients were more likely to be obese and had significantly higher SBP, DBP, ALT, AST, GGT, TC, TG, LDL-C, FBG and lower HDL-C levels (all P<0.001). Moreover, blood cell indices, such as the RBC count, WBC count, PLT levels, and HGB concentration, were all higher in FLD group than those in Non-FLD group (all P<0.001). Gender independent results of participants’ characteristics were similar to the gender stratified results.

**Table 1:** Characteristics of participants in FLD and Non-FLD.

	Men (n = 4137)			Women (n = 4481)			Total (n = 8618)		
	Non-FLD (n = 2682)	FLD (n = 1455)		Non-FLD (n = 3812)	FLD (n = 669)		Non-FLD (n = 3812)	FLD (n = 669)	
**Continuous data**			P^1^			P^1^			P^1^
Age (years)	43.8±12.7	44.8±11.0	0.012	40.8±11.5	48.9±10.7	<0.001	42.04±12.11	46.11±11.04	<0.001
BMI (kg/m²)	23.3±2.6	26.5±2.6	<0.001	21.8±2.6	25.5±3.1	<0.001	22.39±2.73	26.20±2.78	<0.001
WC (cm)	83.6±7.8	92.8±7.2	<0.001	74.6±7.7	85.9±8.2	<0.001	78.33±8.88	90.61±8.15	<0.001
SBP (mmHg)	120.7±15.1	127.6±15.4	<0.001	113.6±15.1	127.6±17.8	<0.001	116.53±15.51	127.60±16.21	<0.001
DBP (mmHg)	76.1±10.8	81.8±11.5	<0.001	70.7±9.8	77.6±10.6	<0.001	72.95±10.57	80.45±11.38	<0.001
FBG (mmol/L)	4.99±0.89	5.48±1.52	<0.001	4.95±0.58	5.49±1.47	<0.001	4.97±0.72	5.48±1.51	<0.001
TG (mmol/L)	1.44±0.97	2.40±1.82	<0.001	1.03±0.64	1.77±1.11	<0.001	1.20±0.82	2.20±1.65	<0.001
TC (mmol/L)	5.21±1.01	5.53±1.14	<0.001	4.98±1.01	5.50±1.11	<0.001	5.07±1.02	5.52±1.13	<0.001
HDL-C (mmol/L)	1.31±0.35	1.15±0.26	<0.001	1.57±0.34	1.35±0.32	<0.001	1.46±0.37	1.21±0.30	<0.001
LDL-C (mmol/L)	3.31±0.87	3.51±0.87	<0.001	3.03±0.79	3.51±0.84	<0.001	3.15±0.84	3.51±0.86	<0.001
RBC (×10^12^/L)	5.15±0.49	5.26±0.48	<0.001	4.56±0.40	4.70±0.40	<0.001	4.80±0.53	5.09±0.53	<0.001
HGB (g/L)	150.54±11.14	153.33±10.42	<0.001	129.43±11.89	132.81±11.43	<0.001	138.15±15.57	146.86±14.36	<0.001
WBC (×10^9^/L)	6.82±1.74	7.41±1.80	<0.001	6.31±1.51	7.04±1.63	<0.001	6.52±1.63	7.29±1.76	<0.001
PLT (×10^9^/L)	230.38±49.41	238.96±50.34	<0.001	254.23±55.62	271.76±58.03	<0.001	244.38±54.42	249.30±55.02	<0.001
ALT (mmol/L)	20.0(15.0, 27.0)	32.0(22.0, 46.0)	<0.001	13.4(10.4, 18.0)	20.0(14.9, 30.3)	<0.001	16.0(11.9, 22.0)	28.0(19.0, 41.7)	<0.001
AST (mmol/L)	22.0(19.0, 25.8)	25.0(21.0, 31.2)	<0.001	19.0(16.3, 22.0)	21.4(18.2, 26.0)	<0.001	20.0(17.0, 23.7)	24.0(20.0, 30.0)	<0.001
GGT (mmol/L)	26.0(19.5, 37.0)	42.0(29.2, 61.0)	<0.001	15.6(12.7, 20.1)	23.0(18.0,32.7)	<0.001	19.0(14.0, 27.7)	35.0(23.5, 55.0)	<0.001
**Category data**			P^2^			P^2^			P^2^
Diabetes n(%)	70(2.6)	136(9.3)	<0.001	42(1.1)	42(6.3)	<0.001	112(1.7)	178(8.4)	<0.001
Overweight n(%)	1074(40)	1245(85.6)	<0.001	699(18.3)	455(68.0)	<0.001	1773(27.3)	1700(80.0)	<0.001
Central obesity n(%)	559(20.8)	941(64.7)	<0.001	904(23.7)	519(77.6)	<0.001	1463(22.5)	1460(68.7)	<0.001

Continuous values are expressed as the means±SD or medians (interquartile ranges).

P values were calculated with covariance analysis for continuous data (P^1^) and Wald Chi square test for category data (P^2^). Age was adjusted in men and women subgroups, while age and sex were adjusted in the total group.

ALT, AST, GGT were logarithmic transformed before analysis.

Abbreviations: BMI, body mass index; WC, waist circumference; SBP, systolic blood pressure; DBP, diastolic blood pressure; FBG, fasting blood glucose; TG, triglyceride; TC, total cholesterol; HDL-C, high-density lipoprotein cholesterol; LDL-C, low-density lipoprotein cholesterol; RBC, red blood cell; HGB, hemoglobin; WBC, white blood cell; PLT, platelet; ALT, alanine aminotransferase; AST, aspartate aminotransferase; GGT, gamma-glutamyl transpeptidase

**Fig 1:**
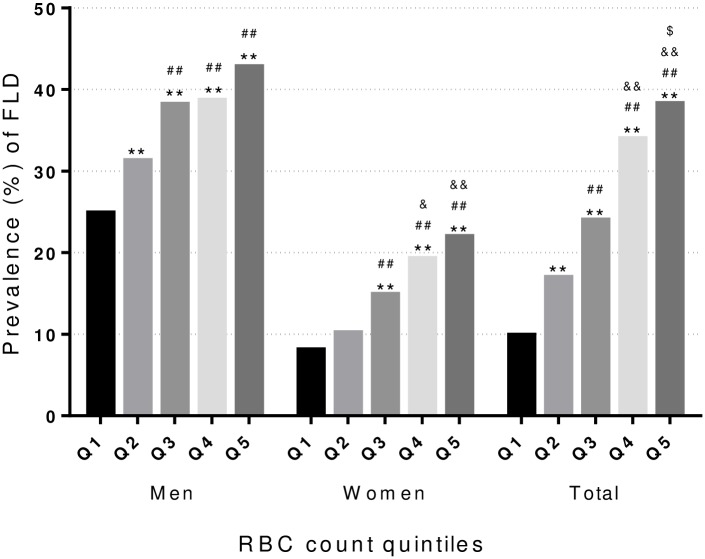
Prevalence of FLD in quintiles of RBC count. *: P<0.05; **: P<0.005 compared with Q1. #: P<0.05; ##: P<0.005 compared with Q2. &: P<0.05; &&: P<0.005 compared with Q3. $: P<0.05; $ $: P<0.005 compared with Q4.

To investigate the association between RBCs and the risk of FLD, all participants were classified into quintiles according to their RBC levels with and without stratification by sex. As shown in Fig 1, the prevalence rate of FLD tended to rise along with the increase of RBC count (all P trend<0.001). The prevalence rates were significantly higher in men than women, ranging from 24.9% to 42.8% in men and 8.1% to 22.0% in women, from Q1 to Q5.

Age-adjusted binary logistic regression analysis showed a significant dose-dependent positive correlation between RBC count and the risk of FLD in men, women and all subjects as a whole (all P trend <0.001) Table 2. The ORs (95% CI) of FLD for Q5 (vs. Q1) for the RBC count was 2.56 (2.06–3.18) for men and 3.69 (2.74–4.98) for women. After further adjustments were made for the indices of waist circumference (WC), fasting blood glucose (FBG), and blood lipids (TG and HDL-C) alone or in combination, the correlations were slightly attenuated, but remained statistically significant (all P trend<0.001). The ORs of Q5 (vs. Q1) (95%CI) decreased to 1.51 (1.17–1.96) in men and 2.27 (1.62–3.19) in women after adjusting for all of these risk factors. The associations between RBCs and FLD risk are stronger in women (OR = 3.69, 95%CI 2.74–4.98 in the highest quintile) than in men (OR = 2.56, 95% CI 2.06–3.18 in the highest quintile); however, sex was not a statistically significant effect modifier (P-interaction by sex = 0.266). Gender independent results also showed that higher RBC count increased the risk of FLD after adjusting for various confounding factors (all P trend<0.001, OR = 2.12, 95%CI 1.64–2.73 in the highest quintiles).

**Table 2:** Odds ratios (ORs) of FLD for quintiles of RBC count.

	Model1 (Age adjusted)	Model2 (Age+WC adjusted)	Model3 (Age+FBG+TG+HDL adjusted)	Model4 (Age+WC+FBG+TG+HDL adjusted)
Men(×10^12^/L)				
Q1(3.07–4.83)	1.00	1.00	1.00	1.00
Q2(4.83–5.05)	1.48(1.19–1.84)	1.09(0.85–1.40)	1.32(1.04–1.67)	1.04(0.80–1.34)
Q3(5.05–5.24)	2.07(1.67–2.57)	1.37(1.07–1.76)	1.74(1.38–2.20)	1.27(0.98–1.64)
Q4(5.24–5.49)	2.18(1.75–2.71)	1.48(1.15–1.91)	1.79(1.41–2.27)	1.35(1.04–2.76)
Q5(5.49–7.49)	2.56(2.06–3.18)	1.67(1.30–2.13)	2.05(1.62–2.59)	1.51(1.17–1.96)
P Trend	<0.001	<0.001	<0.001	0.001
Women(×10^12^/L)				
Q1(3.15–4.28)	1.00	1.00	1.00	1.00
Q2(4.28–4.46)	1.34(0.96–1.87)	1.26(0.88–1.81)	1.35(0.95–1.92)	1.28(0.88–1.85)
Q3(4.46–4.62)	2.28(1.66–3.12)	1.84 (1.30–2.59)	1.96(1.40–2.74)	1.67(1.17–2.38)
Q4(4.62–4.83)	3.04(2.24–4.12)	2.26(1.62–3.15)	2.56(1.85–3.54)	2.03(1.45–2.86)
Q5(4.83–6.64)	3.69(2.74–4.98)	2.63(1.89–3.65)	2.84(2.06–3.91)	2.27(1.62–3.19)
P Trend	<0.001	<0.001	<0.001	<0.001
P-interaction	0.266	0.435	0.585	0.529
Total(×10^12^/L)				
Q1(3.07–4.41)	1.00	1.00	1.00	1.00
Q2(4.42–4.68)	1.77(1.44–2.17)	1.53(1.21–1.92)	1.55(1.25–1.93)	1.41(1.12–1.78)
Q3(4.69–4.95)	2.32(1.89–2.85)	1.67(1.33–2.11)	1.86(1.50–2.31)	1.47(1.16–1.87)
Q4(4.96–5.28)	3.41(2.75–4.21)	2.23(1.74–2.84)	2.65(2.11–3.33)	1.94(1.51–2.50)
Q5(5.29–7.49)	4.30(3.47–5.33)	2.52(1.96–3.22)	3.09(2.45–3.88)	2.12(1.64–2.73)
P Trend	<0.001	<0.001	<0.001	<0.001

ORs of FLD for quintiles of RBC count were calculated with binary logistic regression analyses in different models, with Q1 as the reference.

Models: Model1, adjusted for age; Model2, adjusted for age and WC; Model3, adjusted for age, FPG, TG and HDL; Model4, adjusted for age, WC, FPG, TG and HDL. Sex was also adjusted in all the models of the total group.

P-interaction, interaction between sex and quintiles of RBC count.

**Fig 2:**
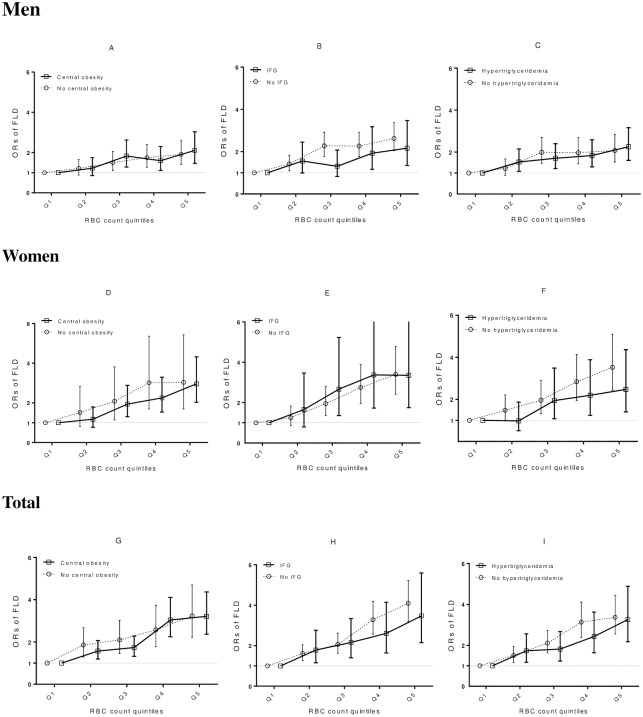
ORs of FLD based on RBC quintiles stratified by classical risk factors for FLD. Results in men subgroup (A, B, C), women subgroup(D, E, F) and the total group (G, H, I) were presented. Stratified factors are central obesity (A, D, G), IFG (B, E, H) and hypertriglyceridemia (C, F, I). ORs with 95% CI are presented compared with the reference Q1 of RBCs. ORs are based on the reference Q1 within each group. OR, odds ratio; IFG, impaired fasting glucose.

We further examined the correlation of RBC quintiles with the risk of FLD stratified by various FLD risk factors (WC, IFG and TG) using binary logistic regression analysis. As shown in Fig 2, similar associations of increased RBCs with FLD risk were seen between subjects with and without central obesity, IFG, and hypertriglyceridemia. No significant interactions were detected between RBCs and these risk factors for FLD.

## Discussion

This study was a large scale survey to explore the correlation between the RBC count and risk of FLD in southern Chinese adults. We found that the RBC count was independently associated with the risk of FLD. First, subjects in FLD had higher RBC counts compared with Non-FLD. Second, subjects in higher quintiles of RBCs had a higher prevalence and ORs for FLD (P trend<0.001), with ORs of 2.56–1.51 (P<0.001) and 3.69–2.27 (P<0.001) in Q5 (vs Q1), when adjusted for different covariates in men and women, respectively. Similar results were found in gender independent analyses. Furthermore, after stratifying the FLD risk factors, similar associations of increasing RBC quintiles with FLD risk were observed in sub-stratified groups.

Previous studies examining the association between RBC counts and FLD are rare, and the outcomes remain inconclusive. Jiang YZ *et al*. investigated 977 participants and reported that the RBC count was independently and positively associated with NAFLD prevalence and risk [12]. The same results were found in another study [24]. In our present study, the prevalence of FLD was higher in men (35.17%) than that in women (24.65%), however, the prevalence and ORs of FLD are dose-dependently increase by RBC count before and after adjusting for various confounders in men, women and combined. The similarity of gender dependent and independent results strengthen that higher RBC count is an independent risk factor for FLD.

Another study, including 2000 subjects who received annual physical examinations, also found that both lean and overweight-obese NAFLD subjects were associated with higher RBC counts [25]. However, Danny Issa *et al*. contended that the RBC count showed no significant association with the histologic features of NAFLD [18]. This inconsistency might be attributed to small sample sizes in the previous studies, different research designs and analyses methods, or inclusion of subjects from different regions.

The findings in this study can provide some novel insights into the potential prognosis of FLD patients using RBC counts. Our large-scale cross-sectional study showed that subjects with higher RBC counts had significantly higher prevalence and risk of FLD in both gender. Several cross-sectional studies and one longitudinal study have demonstrated that the RBC count is positively associated with MetS and different components of MetS [26–28]. As FLD has been widely regarded as a phenotype of MetS in the liver and because obesity, hyperlipidemia, diabetes are all risk factors for FLD, this evidence may indirectly lend support to the relationship between the RBC count and FLD.

The mechanism underlying the association between the RBC count and FLD remains elusive. One of the possible explanations may be the compensatory erythropoiesis due to the impairment of the liver metabolism and detoxification function in FLD status. RBCs are well equipped with antioxidant systems involved with tissue protection and regulation of cardiovascular homeostasis, and enhanced erythropoiesis may help remedy liver function. Besides, erythropoiesis might be enhanced in FLD as iron overload and hyperferritinemia have been reported to be associated with NAFLD. Iron is essential to the generation of hemoglobin and erythrocyte, which may lead to increased RBCs in circulation [29–31]. Furthermore, oxidative stress could enhance compensatory erythropoiesis in chronic inflammatory diseases, such as atherosclerosis, coronary artery disease, metabolic syndrome and FLD. The main physiological roles of RBCs are not only transporting oxygen and carbon dioxide but also scavenging reactive oxygen and nitrogen species. As FLD is frequently accompanied with inflammation and oxidative damage [32, 33], and RBC indices have been suggested to be associated with inflammatory markers [7, 34], generation of RBCs may be increased. Additionally, the capacity of spleen and liver for scavenging RBCs might be damaged in FLD condition, leading to further increase of RBC count. However, the exact and detailed mechanism still needs further study in animal and cell models, or studies with fatty liver biopsy samples to confirm the correlation between RBC count and FLD.

Some limitations are present in our study. First, it was not a prospective study, whether the variation in RBC levels is just a biomarker of FLD or a cause-and-effect relationship exists still remains unclear. Prospective studies or therapeutic interventions are needed to investigate whether change in RBC count would affect occurrence and development of FLD in the future. Second, FLD patients were diagnosed with abdominal ultrasonography, which could not accurately diagnose FLD and further stages of the spectrum of fatty liver, steatohepatitis and fibrosis; therefore, ultrasonography was unable to detect the correlation of RBC levels with different stages of FLD in this study. Third, the difference in RBC count between FLD patients and healthy subjects was relatively small, and the RBC counts in many FLD patients were within the normal range (4.0–5.5×10^12^/L for men, 3.5–5.0×10^12^/L for women), making it difficult to identify a definite cut-off for predicting FLD. Nonetheless, our results indicated that subjects with high RBC counts, especially combined with higher values for WC, were at a higher risk of FLD.

In summary, the RBC count was higher in FLD subjects than in non-FLD subjects and was independently associated with the prevalence of FLD. The gender dependent and independent results provides evidence in support of using RBC count as a valuable predictor of FLD; meanwhile, large-scale longitudinal cohort studies are needed to confirm the present findings. Further studies are needed to elucidate the mechanisms responsible for increased RBC counts among individuals with fatty liver disease.

## Supporting information

S1 Table Supplemental dataset. (XLSX)

## Abbreviations

ALT: alanine aminotransferase
AST: aspartate aminotransferase
BMI: body mass index
CI: confidential interval
DBP: diastolic blood pressure
FBG: fasting blood glucose
FLD: fatty liver disease
GGT: gamma-glutamyl transpeptidase
HDL-C: high-density lipoprotein cholesterol
HGB: hemoglobin
LDL-C: low-density lipoprotein cholesterol
OR: odds ratio
PLT: platelet
RBC: red blood cell
SBP: systolic blood pressure
TC: total cholesterol
TG: triglyceride
WBC: white blood cell
WC: waist circumference
